# Various impacts of driver mutations on the PD-L1 expression of NSCLC

**DOI:** 10.1371/journal.pone.0273207

**Published:** 2022-08-18

**Authors:** Cheng-Hsiang Chu, Yen-Hsiang Huang, Po-Hsin Lee, Kuo-Hsuan Hsu, Kun-Chieh Chen, Kang-Yi Su, Sung-Liang Yu, Jeng-Sen Tseng, Tsung-Ying Yang, Gee-Chen Chang

**Affiliations:** 1 Division of Chest Medicine, Department of Internal Medicine, Taichung Veterans General Hospital, Taichung, Taiwan; 2 Division of Pulmonary Medicine, Department of Internal Medicine, Chung Shan Medical University Hospital, Taichung, Taiwan; 3 School of Medicine, Chung Shan Medical University, Taichung, Taiwan; 4 Institute of Biomedical Sciences, National Chung Hsing University, Taichung, Taiwan; 5 College of Medicine, National Yang Ming Chiao Tung University, Taipei, Taiwan; 6 Ph.D. Program in Translational Medicine, National Chung Hsing University, Taichung, Taiwan; 7 Rong Hsing Research Center For Translational Medicine, National Chung Hsing University, Taichung, Taiwan; 8 Division of Critical Care and Respiratory Therapy, Department of Internal Medicine, Taichung Veterans General Hospital, Taichung, Taiwan; 9 Institute of Medicine, Chung Shan Medical University, Taichung, Taiwan; 10 Department of Clinical Laboratory Sciences and Medical Biotechnology, College of Medicine, National Taiwan University, Taipei, Taiwan; 11 Department of Laboratory Medicine, National Taiwan University Hospital, Taipei, Taiwan; 12 Institute of Medical Device and Imaging, College of Medicine, National Taiwan University, Taipei, Taiwan; 13 Graduate Institute of Pathology, College of Medicine, National Taiwan University, Taipei, Taiwan; 14 Graduate Institute of Clinical Medicine, College of Medicine, National Taiwan University, Taipei, Taiwan; 15 Department of Post-Baccalaureate Medicine, College of Medicine, National Chung Hsing University, Taichung, Taiwan; 16 Department of Life Sciences, National Chung Hsing University, Taichung, Taiwan; Osmania University, Hyderabad, India, INDIA

## Abstract

We aimed to evaluate whether different driver mutations have varying impacts on the programmed cell death-ligand 1 (PD-L1) expression of non-small cell lung cancer (NSCLC), and whether the prognostic roles of PD-L1 amongst our patients were divergent. This was a single-institute study that included patients with NSCLC. Six driver mutations, PD-L1 status, and the outcomes of treatment were assessed. A total of 1,001 NSCLC patients were included for analysis. Overall, the PD-L1 positive (TPS ≥ 1%) and strong positive (TPS ≥ 50%) rates were 52.2% and 17.3%, respectively. As compared with wild type lung adenocarcinoma, *EGFR*-mutant and *HER2*-mutant patients had similarly low PD-L1 and strong PD-L1 positive rates. *BRAF*-mutant patients had numerically higher PD-L1 and strong PD-L1 positive rates. Patients with fusion mutation (*ALK* and *ROS1*) (aOR 2.32 [95% CI 1.10–4.88], P = 0.027 and 2.33 [95% CI 1.11–4.89], P = 0.026), *KRAS* mutation (aOR 2.58 [95% CI 1.16–5.75], P = 0.020 and 2.44 [95% CI 1.11–5.35], P = 0.026), and non-adenocarcinoma histology (aOR 2.73 [95% CI 1.72–4.34], P < 0.001 and 1.93 [95% CI 1.13–3.30], P = 0.016) all had significantly higher PD-L1 and strong PD-L1 positive rates. A trend towards longer survival was noted in *ROS-1* rearranged and *KRAS*-mutant patients with strong PD-L1 expression who had received crizotinib and chemotherapy, respectively. In conclusion, individual driver mutations had various impacts on the PD-L1 expression of NSCLC patients. The prognostic role of PD-L1 may also be divergent amongst patients harboring different driver mutations.

## Introduction

Lung cancer is the leading cause of cancer related death worldwide [1]. In addition to chemotherapy and targeted therapy, immunotherapy has emerged as a novel and effective treatment option for patients with advanced non-small cell lung cancer (NSCLC) and the treatment choices are dependent upon each patient’s characteristics. Hence, the biomarker assessment plays a critical role in the management of NSCLC patients, which includes histological types, driver mutation status, and immunological expression status [2, 3]. The interaction between biomarkers could possibly be more complex and may serve as a prognostic factor in treatment [4, 5].

Programmed cell death-ligand 1 (PD-L1) expression status is an important biomarker for selecting patients to receive immunotherapy, particularly in the first-line setting [6, 7]. Moreover, studies have suggested that PD-L1 status can be a useful predictor of the efficacy of certain targeted therapies [4, 8, 9]. Currently, the PD-L1 status is one of the mandatory biomarkers suggested in the clinical guidelines regarding metastatic NSCLC management [2, 10] and it is required to understand the association between PD-L1 expression and patients’ clinicopathological features, including the status of driver mutations. Many studies have addressed this issue, but the results were not consistent [11–15]. Our prior study recognized that patients without actionable driver mutations were more likely to present positive and strong positive PD-L1 [13]. However, the impact of individual driver mutations on the PD-L1 expression is still not yet well-documented.

Although the checkpoint blockade therapy is indicated majorly in patients who are *epidermal growth factor receptor* (*EGFR*) and *anaplastic lymphoma kinase* (*ALK*)-wild type [2, 10], its role amongst patients harboring other uncommon driver mutations are still uncertain. Furthermore, one’s PD-L1 status could predict the outcome of targeted therapy for *EGFR*-mutant and *ALK*-positive patients [4, 8, 9]. Hence, it remains worthwhile to explore the interaction between PD-L1 expression and driver mutations in the entire NSCLC population. Herein, we have conducted a single-institute study with a larger cohort in order to evaluate the interaction between six oncogenic drivers and the PD-L1 status amongst patients with NSCLC, and focused on whether individual oncogenic drivers have different impacts on the PD-L1 expression.

## Materials and methods

### Patients

We included lung cancer patients who had been diagnosed and treated at Taichung Veterans General Hospital (TCVGH) between December 2009 and August 2020. To be eligible for participation in the study, patients were required to have cytologically or pathologically confirmed lung cancer, clear clinical follow-up data, and adequate tumor specimens for PD-L1 assays and/or driver mutation testing. Of them, driver mutation analysis, which included six common oncogenic drivers, was mandatory for patients with adenocarcinoma histology. Patients were excluded if they had lung malignancy of a doubtful origin, other active tumors, or incomplete data records.

This study was approved by the institutional review board of Taichung Veterans General Hospital (IRB No.: CF20175 & CF20176). Written informed consents for clinical data records, genetic and immunological testing were obtained from all patients.

### Data records and response evaluation

Clinical data for analysis included patients’ age, gender, Eastern Cooperative Oncology Group performance status (ECOG PS), tumor stage, smoking status, driver mutation status, histological types, and PD-L1 expression status. The TNM (tumor, node, and metastases) staging was evaluated according to the 8th edition of the American Joint Committee for Cancer (AJCC) staging system [16]. One-dimensional measurements as determined by the Response Evaluation Criteria in Solid Tumors (RECIST) version 1.1 were used in this study to evaluate the response of treatment [17].

### Driver mutation analysis

Six oncogenic drivers, including *EGFR*, *Kirsten rat sarcoma viral oncogene homolog* (*KRAS*), *v-raf murine sarcoma viral oncogene homolog B* (*BRAF*), *human epidermal growth factor receptor 2* (*HER2*), *ALK*, and *ROS1* were tested. *EGFR*, *KRAS*, *BRAF*, and *HER2* mutations were assessed using matrix-assisted laser desorption ionization-time of flight mass spectrometry (MALDI-TOF MS), *ALK* fusion mutation was tested with a fully automated IHC assay (Ventana IHC, Ventana, Tucson, AZ) using the pre-diluted Ventana anti-ALK (D5F3) Rabbit monoclonal primary antibody, and *ROS1* fusion mutation was determined by fluorescent in situ hybridization (FISH) as previously described [18].

### PD-L1 assay

Formalin-fixed, paraffin embedded samples, whether they were tumor tissues or cell blocks from cytological specimens were collected for PD-L1 IHC assay. All viable tumor cells on the slide prepared as 4-mm-thick with the hematoxylin and eosin–stained sections were evaluated. The presence of at least 100 viable tumor cells was required for the specimen to be considered adequate for quantification of PD-L1 expression. PD-L1 status was accessed by the Ventana PD-L1 SP263 assay as previously described [13]. All the slides were peer reviewed by two pathologists, and the results have been concurred in an intradepartmental consensus meeting.

### Statistical methods

Univariate analyses of PD-L1 expression were performed by the Fisher’s exact test. The Kaplan–Meier method was used to estimate the survival time. Difference in survival time was analyzed by the log-rank test. The logistic regression model was used for multivariate analyses of PD-L1 expression. In the stepwise procedure, the significant levels for entry and removal were 0.05 and 0.10, respectively. All statistical tests were carried out using SPSS 15.0 (SPSS Inc., Chicago, IL, USA). Two-tailed tests and P values <0.05 for significance were implemented.

## Results

### Patient characteristics

A total of 1,001 NSCLC patients were included for analysis. Patients’ characteristics were summarized in Table 1. The median age was 64 years. Of them, 481 patients (48.1%) were female, while 581 patients (58.0%) were non-smokers. Adenocarcinoma accounted for the major histological types (79.0%) and 549 patients (54.8%) had metastatic disease.

**Table 1:** Demographic data and patients’ characteristics.

Characteristics	N = 1,001 NSCLC patients
Age, median (range), years	64 (22–97)
Gender, n (%)	
Female	481 (48.1)
Male	520 (51.9)
Smoking status, n (%)	
Non-smokers	581 (58.0)
Smokers	420 (42.0)
Histological types, n (%)	
Adenocarcinoma	791 (79.0)
Non-adenocarcinoma^#^	210 (21.0)
Tumor stage, n (%)	
Stage I-III	452 (45.2)
Stage IV	549 (54.8)

NSCLC, non-small cell lung cancer.

*Includes patients with adenocarcinoma mixed with other histological types.

### PD-L1 expression and driver mutation status

**Fig 1:**
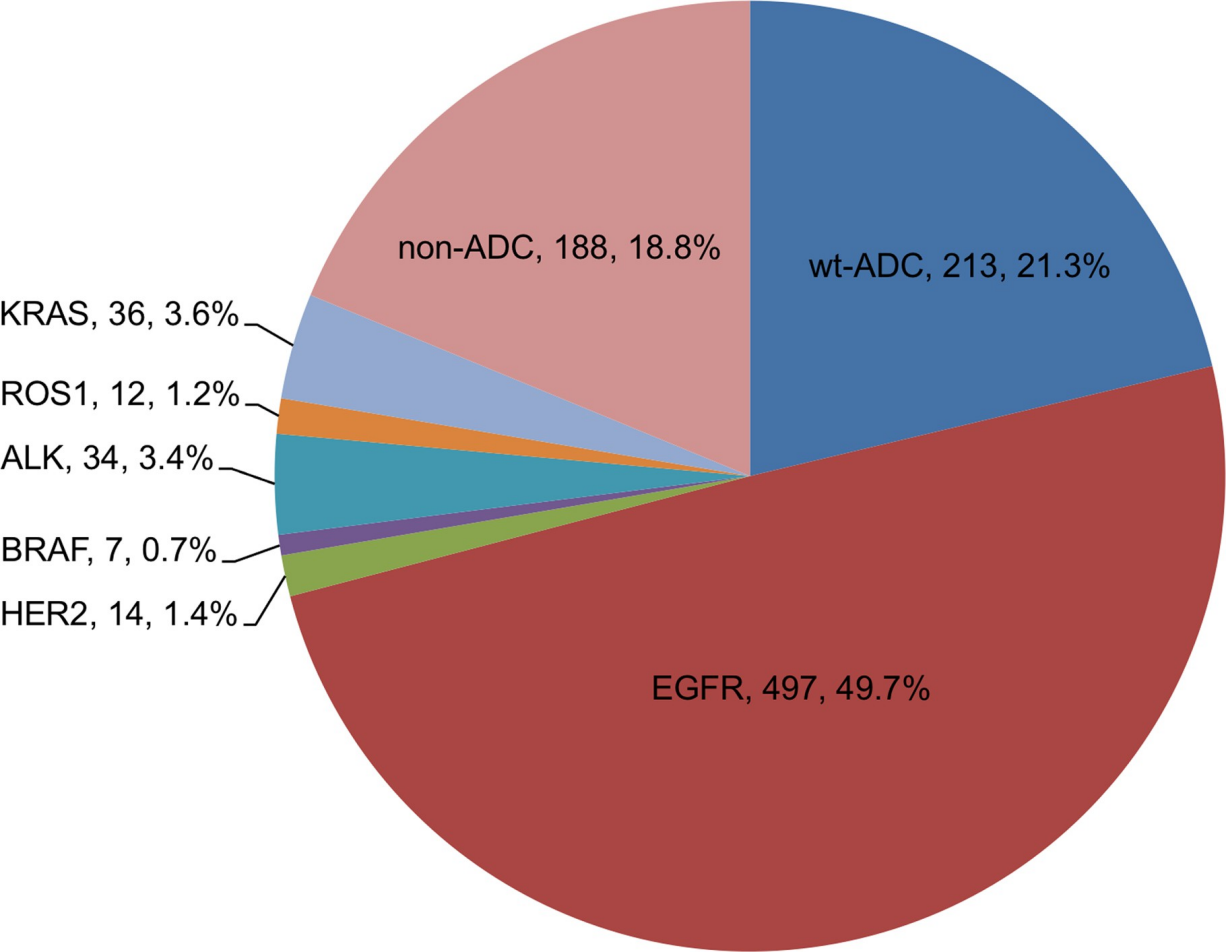
Histology and genotype distribution of NSCLC. ADC, adenocarcinoma; wt, *EGFR* and *ALK*-wild type.

Fig 1 summarizes the distribution of histological types and driver mutation status. *EGFR* mutation accounted for the majority of genetic alteration amongst our NSCLC population (49.7%), followed by *KRAS* mutation (3.6%), *ALK* fusion (3.4%), *HER2* mutation (1.4%), *ROS1* rearrangement (1.2%), and *BRAF* mutation (0.7%). Of note, a total of 19 non-adenocarcinoma patients harbored *EGFR* mutations, including 12 with squamous cell carcinoma, 4 with carcinoma not otherwise specified, 2 with mixed histology, and 1 with sarcomatoid carcinoma. Within those 19 patients, 13 patients (68.4%) were non-smokers. There were 3 non-adenocarcinoma patients harboring *KRAS* mutation (2 with squamous cell carcinoma and 1 with carcinoma not otherwise specified). Hence, 213 patients (21.3%) and 188 patients (18.8%) were categorized as *EGFR*/*ALK*-wild type adenocarcinoma and non-adenocarcinoma groups, respectively.

In the case of PD-L1 expression status, 478 patients were PD-L1 negative, while 350 patients had a PD-L1 tumor proportion score (TPS) between 1–50% and 173 patients with PD-L1 had a TPS ≥ 50%. Overall, the PD-L1 positive rate was 52.2% and the PD-L1 strong positive rate was 17.3%. Univariate analyses of the association between patients’ characteristics and PD-L1 expression were summarized in Table 2. Patients who were at a younger age, male, with a smoking history, and non-adenocarcinoma histology were more likely to have a positive PD-L1 expression (P value = 0.007, 0.027, < 0.001, and < 0.001, respectively), while patients with a smoking history and non-adenocarcinoma histology were more likely to have a strong PD-L1 expression (P = 0.027 and < 0.001, respectively). These factors were integrated in multivariate analysis in order to adjust the impact of driver mutations.

**Table 2:** Univariate analysis of patient’s characteristics and PD-L1 expression.

Characteristics	PD-L1 ≥ 1%	P value*	PD-L1 ≥ 50%	P value*
Age, n (%)		0.007		0.261
<60 years	215 (57.8)		71 (19.1)	
≥60 years	308 (49.0)		102 (16.2)	
Gender, n (%)		0.027		0.209
Female	233 (48.5)		75 (15.6)	
Male	290 (55.7)		98 (18.8)	
Smoking status, n (%)		<0.001		0.027
Non-smokers	268 (46.1)		87 (15.0)	
Smokers	255 (60.7)		86 (20.5)	
Histological types, n (%)		<0.001		<0.001
Adenocarcinoma	373 (47.2)		115 (14.5)	
Non-adenocarcinoma^#^	150 (71.4)		58 (27.6)	
Tumor stage, n (%)		0.253		0.503
Stage I-III	227 (50.2)		74 (16.4)	
Stage IV	296 (53.9)		99 (18.0)	

PD-L1, programmed death ligand 1.

^#^Includes patients with adenocarcinoma mixed with other histological types.

*By Fisher’s exact test.

### Impact of various driver mutations on the expression of PD-L1

**Fig 2:**
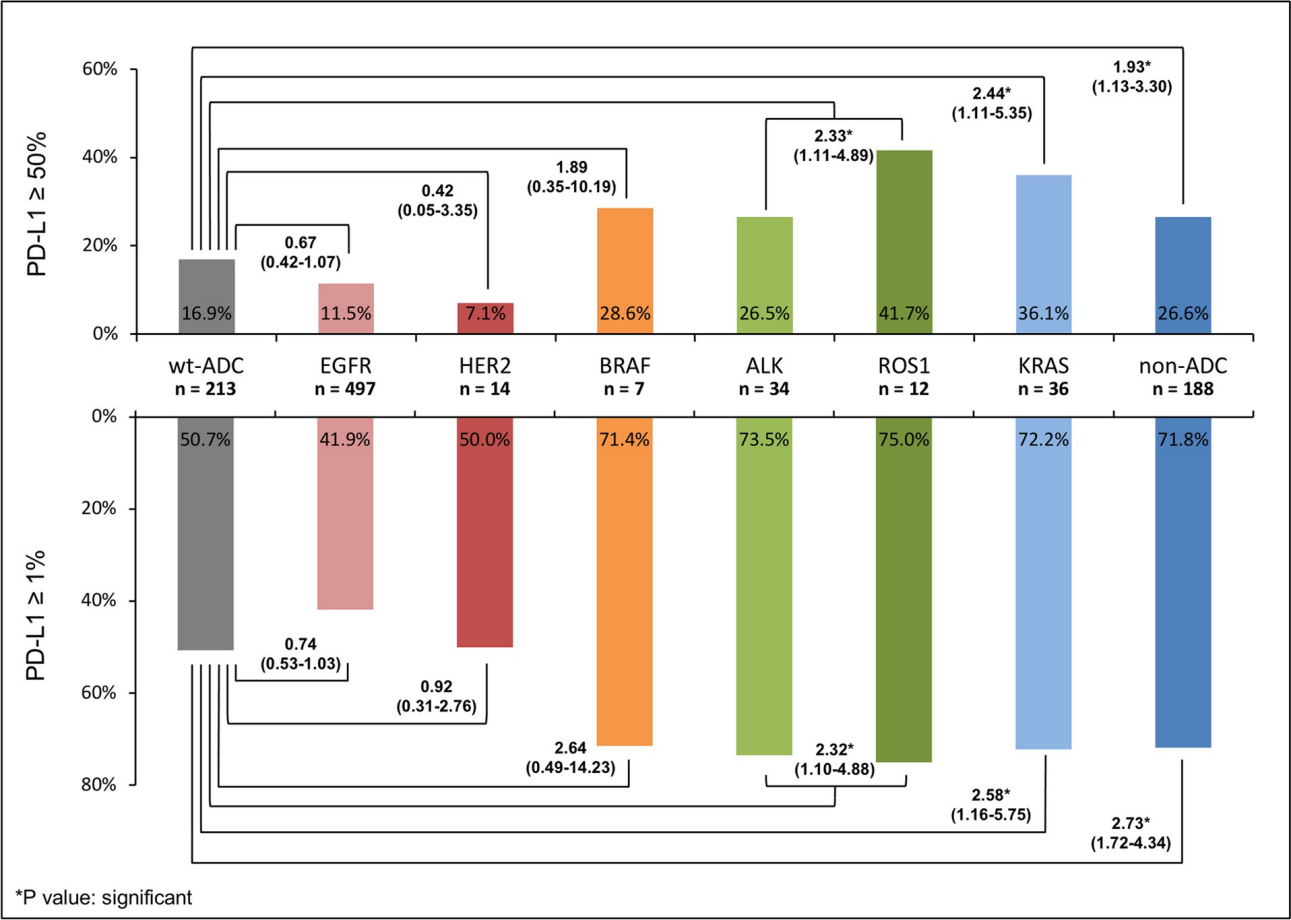
Various impacts of driver mutations on the PD-L1 expression of NSCLC.

The results evaluating the impact of various driver mutations on the expression of PD-L1 were shown in Table 3 and Fig 2. Here, *EGFR*/*ALK*-wild type lung adenocarcinoma was set as the reference for comparison. Data were presented by the percentage of PD-L1 positivity and adjusted odds ratio.

**Table 3:** Impact of driver mutation status on the PD-L1 expression.

	PD-L1 (+)	P value*	PD-L1 strong (+)	P value*
wt-ADC^#^	1.00 (Reference)	N/A	1.00 (Reference)	N/A
*EGFR*	0.74 (0.53–1.03)	0.078	0.67 (0.42–1.07)	0.094
*HER2*	0.92 (0.31–2.76)	0.886	0.42 (0.05–3.35)	0.413
*BRAF*	2.64 (0.49–14.23)	0.258	1.89 (0.35–10.19)	0.461
*ALK & ROS1*	2.32 (1.10–4.88)	0.027	2.33 (1.11–4.89)	0.026
*KRAS*	2.58 (1.16–5.75)	0.020	2.44 (1.11–5.35)	0.026
non-ADC	2.73 (1.72–4.34)	<0.001	1.93 (1.13–3.30)	0.016

PD-L1, programmed death ligand 1; wt, wild type; ADC, adenocarcinoma.

*By logistic regression model; data presented by adjusted odds ratio.

^#^Denotes patients with both *EGFR* and *ALK* wild type lung adenocarcinoma.

As compared with *EGFR*/*ALK*-wild type lung adenocarcinoma patients, *EGFR*-mutant (aOR 0.74 [95% CI 0.53–1.03], P = 0.078 and 0.67 [95% CI 0.42–1.07], P = 0.094) and *HER2*-mutant (aOR 0.92 [95% CI 0.31–2.76], P = 0.886 and 0.42 [95% CI 0.05–3.35], P = 0.413) patients had a similarly low PD-L1 and strong PD-L1 positive rate. *BRAF*-mutant patients had a numerically higher PD-L1 and strong PD-L1 positive rate (aOR 2.64 [95% CI 0.49–14.23], P = 0.258 and 1.89 [95% CI 0.35–10.19], P = 0.461). Patients with fusion mutation (*ALK* and *ROS1*) (aOR 2.32 [95% CI 1.10–4.88], P = 0.027 and 2.33 [95% CI 1.11–4.89], P = 0.026), *KRAS* mutation (aOR 2.58 [95% CI 1.16–5.75], P = 0.020 and 2.44 [95% CI 1.11–5.35], P = 0.026), and non-adenocarcinoma histology (aOR 2.73 [95% CI 1.72–4.34], P < 0.001 and 1.93 [95% CI 1.13–3.30], P = 0.016) all had a significantly higher PD-L1 and strong PD-L1 positive rate.

In the non-adenocarcinoma group (n = 188), a strong PD-L1 expression was seen in 26.6% of in patients with squamous cell carcinoma (n = 139) and in 26.5% of the non-squamous cell carcinoma population (n = 49), respectively. P value was not significant (= 1.000).

### Impact of PD-L1 expression status on the outcome of treatment

Since driver mutations have varying impacts on the expression of PD-L1, the prognostic role of PD-L1 amongst patients harboring with different driver mutations may also be varying.

Our prior studies have evaluated the role of PD-L1 on the outcome of advanced *EGFR*-mutant and *ALK*-positive NSCLC patients [4, 8]. In the case of *EGFR* mutation, a strong PD-L1 expression was associated with a worse progression-free survival (PFS), overall survival (OS), and the resistance to EGFR-TKI treatment. With regards to *ALK* fusion, the OS could be sorted by the *ALK* variants and PD-L1 expression. Within them, patients with non-V3a/b subtype and positive PD-L1 showed a trend towards a longer OS.

Herein, we further evaluated the impacts of PD-L1 expression on the outcome of patients harboring other uncommon driver mutations, with the results being summarized in Table 4. Six out of 12 *ROS-1*-positive patients were treated with crizotinib, with 3 of them showing strong PD-L1 expression. Due to the limited case numbers, we showed the details of the patients’ characteristics and their treatment outcomes in Table 4. Four patients achieved an objective response to the crizotinib treatment, one patient had stable disease, while one patient did not have any measurable lesion. Regarding survival analysis, PD-L1 status did not influence the PFS (11.5 months [95% CI 0.3–22.7] vs. 8.2 months [95% CI NR-NR]; P = 0.364), whereas there was a trend towards a longer OS in patients with a strong PD-L1 expression (NR [95% CI NR-NR] vs. 11.5 months [95% CI NR-NR]; P = 0.083).

**Table 4:** PD-L1 expression and the outcome of advanced NSCLC treatment.

***ROS1* fusion and *BRAF* mutation: Targeted therapy**											
No.	Age	Gender	PS	Mutation	PD-L1	Regimen (line)^$^		Res.	PFS^#^		OS^#^
1	50	Male	1	*ROS1*	5%	Crizotinib (1)		PR	7.9*		7.9*
2	32	Female	1	*ROS1*	1%	Crizotinib (2)		SD	4.2		11.5
3	29	Male	1	*ROS1*	50%	Crizotinib (2)		PR	11.5		18.4*
4	51	Female	2	*ROS1*	90%	Crizotinib (2)		PR	4.5		13.6
5	50	Female	1	*ROS1*	90%	Crizotinib (2)		NA	15.9*		15.9*
6	69	Female	2	*ROS1*	0%	Crizotinib (3)		PR	8.2		10.3*
7	57	Male	1	*BRAF*	5%	DAB/TRA (1)		PD	2.7		14.2*
8	64	Female	2	*BRAF*	30%	DAB/TRA (1)		NA	8.9*		8.9*
***KRAS* mutation: Platinum doublet chemotherapy**											
PD-L1		Patient No.		PFS (m)		P value^&^	OS (m)			P value^&^	
≥ 50%		10		1.7 (0.0–4.4)		0.720	NR (NR-NR)			0.189	
< 50%		10		3.9 (0.5–7.3)			5.3 (1.4–9.2)				
***HER2* mutation: Platinum doublet chemotherapy**											
PD-L1		Patient No.		PFS (m)		P values^&^	OS (m)			P value^&^	
≥ 1%		4		4.9 (0.0–13.8)		0.779	18.2 (NR-NR)			0.988	
Negative		4		2.7 (0.0–13.4)			26.9 (NR-NR)				

PS, Eastern Cooperative Oncology Group performance status; PD-L1, programmed cell death-ligand 1; Res., objective response; PFS, progression-free survival; OS, overall survival; PR, partial response; SD, stable disease; PD, progressive disease; NA, no targeted lesion, not applicable; DAB/TRA: dabrafenib + trametinib; NR, not-reached.

^$^Denotes the regimen and the line of this treatment prescribed.

^#^Presented in months.

*Denotes “no progression yet” and “still alive” for PFS and OS, respectively.

^&^By log-rank test.

There were only two patients with *BRAF* mutation receiving the corresponding targeted therapy. The details of these patients’ characteristics were shown in Table 4. Within them, one patient with a 5% PD-L1 expression was primary resistant to targeted therapy, while the other patient with a 30% PD-L1 expression had been well controlled through the use of dabrafenib plus trametinib, with the treatment ongoing.

Since there was no approved standard targeted therapy for patients with *KRAS* and *HER2* mutation, we evaluated the efficacy of platinum doublet in our patients. Twenty out of 36 *KRAS*-mutant patients underwent platinum doublet chemotherapy. The median age was 62 years (range 47–92). Of those, 4 patients were non-smokers, 5 patients were female, and 17 patients had baseline ECOG PS 0–1. Adenocarcinoma accounted for the majority of histological types, with the exception of 2 patients with squamous cell carcinoma and 1 patient with carcinoma, not otherwise specified. The PD-L1 status was negative, weak positive, and strong positive in 7, 3, and 10 patients, respectively. With regards to the partner of platinum chemotherapy, 2 patients received gemcitabine, 2 patients received paclitaxel, while all the others underwent pemetrexed treatment. In the response evaluation, 5 patients achieved partial response, 4 patients had stable disease, 9 patients had progressive disease, while 2 patients did not have any measurable lesion. The median PFS and OS were 3.4 months (95%CI 0.5–6.2) and 11.8 months (95% CI 0.0–26.4), respectively. PD-L1 positivity did not influence the PFS nor OS significantly (P = 0.318 and 0.658, respectively). However, patients with a strong PD-L1 expression had a numerically longer OS (NR vs. 5.3 months [95% CI 1.4–9.2]) (Table 4 and S1 Fig).

Eight out of 14 *HER2*-mutant patients underwent platinum doublet chemotherapy. Their median age was 55 years (range 39–74). Of those, 6 patients were non-smokers, 3 patients were female, all patients were adenocarcinoma in histology, and all of them had baseline ECOG PS 0–1. The PD-L1 status was negative, weak positive, and strong positive in 4, 3, and 1 patient, respectively. With regards to the partner of platinum chemotherapy, 1 received paclitaxel, while all the others underwent pemetrexed treatment. In the response evaluation, 3 patients achieved partial response, 1 had stable disease, 3 had progressive disease, and 1 did not have any measurable lesion. The median PFS and OS were 4.9 months (95% CI 0.0–14.4) and 26.9 months (95% CI NR-NR), respectively. PD-L1 positivity did not influence the PFS and OS significantly (P = 0.779 and 0.988, respectively).

## Discussion

In spite of high mortality rate, the outcome of lung cancer patients continues to improve. The most important advancement of lung cancer management in a recent decade has been in the way of personalized therapy [3]; of which the assessment of biomarkers plays a crucial role in the decision regarding treatment options. Currently, clinical practice guidelines for NSCLC have suggested more and more biomarkers, particularly oncogenic drivers, to be mandatory in the handling of patient management [2, 10, 19]. However, prospective studies regarding the efficacy of checkpoint inhibitors have usually evaluated only the *EGFR* and *ALK* mutation status [6, 7, 20]. It is still not well-documented what the impact of other uncommon driver mutations on the PD-L1 expression is, and the prognostic role of PD-L1 status on the outcome of treatment of these patients. Herein, we conducted a single institute study with a larger cohort in order to address this issue, which may offer the advantages of better homogeneity in the tissue processing, the operating procedures of biomarker assays, as well as the interpretation of results. Our results suggested that different driver mutations have various impacts on the expression of PD-L1, and that the prognostic role of PD-L1 amongst advanced NSCLC patients with different driver mutations may be also varying.

Recently, several large-scale studies have reported the association between PD-L1 expression and molecular aberrations in NSCLC patients. However, these results were not consistent. Evans evaluated the PD-L1 status with 22C3 assay amongst 10,005 NSCLC patients in England and identified that patients who were *EGFR*-wild type or *ALK*-positive were more likely to present both positive and strong positive PD-L1 [21]. There was no significant association between PD-L1 expression and the *KRAS* mutation. Zheng et al. analyzed 6,295 patients in China with 22C3 assay and suggested that PD-L1 expression was associated with wild type *EGFR* and positive *ALK* fusion [22]. *ROS1* fusion did not influence the PD-L1 expression. Wu et al. reviewed 428 surgically resected lung adenocarcinoma cases [23], where the PD-L1 status was determined by SP142 assay, with the results suggesting that PD-L1 expression was associated with wild type *EGFR*, positive *ALK* fusion, and *KRAS* mutation. However, no significant impact of *ROS1* fusion was found. Karatrasoglou et al. examined 220 patients in Greece and reported that PD-L1 expression was positively correlated with *KRAS* mutation, but there was no significant association with the *EGFR*, *ALK*, *ROS1*, and *BRAF* alterations [11]. In the data analysis procedure, these studies usually compared the PD-L1 expression between patients who were either mutant or wild type in their individual driver genes, which would lead to the overlapping of patients in the reference group. For example, the so-called *EGFR*-wild type population may contain patients who were *KRAS*-mutant; hence, the reference cohort would be heterogeneous, possibly resulting in some biases of data interpretation. In the present study, all patients in the reference group were both *EGFR* and *ALK* wild type, with no patient having any other known uncommon driver gene mutations. The impacts of individual driver genes were evaluated using the same reference cohort. More importantly, there was no overlapping of patients between all the comparison groups. Herein, we suggested that *EGFR*-mutant and *HER2*-mutant patients had a similarly low PD-L1 expression rate to that of wild-type adenocarcinoma patients. Patients who were *KRAS*-mutant, and having a non-adenocarcinoma histology, as well as those with fusion mutation were significantly associated positive PD-L1 and strong PD-L1 expression. Patients with *BRAF* mutation also had a numerically higher PD-L1 expression than wild type adenocarcinoma patients.

The analysis of uncommon driver mutations is usually limited by patient numbers, particularly those with a prevalence less than 3%. Herein, we analyzed *ALK* fusion and *ROS1* fusion together because the two fusion alterations have similar structures, crizotinib sensitivity, and resistant mechanisms [24]. When we analyzed individual genes, both *ALK* and *ROS1* fusion patients still had a higher PD-L1 positive rates than wild type adenocarcinoma patients. A previous study has also suggested that *ROS1* rearrangement was associated with high PD-L1 expression [25]. The association between *HER2* mutation and PD-L1 expression has been far less investigated [26]. In our study, patients with *HER2* mutation had a similarly low PD-L1 expression rate with those in the *EGFR*-mutant group. Since *HER2* and *EGFR* both belong to the ErbB family and share similar downstream pathways [27], further researches could evaluate whether these two mutations play a similar role in immunogenicity, PD-L1 expression, and tumor microenvironment. *BRAF* mutation is a rare genetic alteration in lung cancer [18]. Although we demonstrated a numerically higher PD-L1 positive rate of *BRAF*-mutant patients, it was not statistically significant. A study by Dudnik et al., which evaluated PD-L1 expression in 39 *BRAF*-mutant NSCLC patients, also suggested that *BRAF* mutation is associated with a high level of PD-L1 expression regardless of being the V600E subtype or not [28]. Once again, further research is still required.

In both the present study and our prior works, we have tried to evaluate the prognostic role of PD-L1 in patients with various driver mutations. The most confident results came from the patients with *EGFR*-mutation because our study and several other investigations all suggested that strong PD-L1 expression was associated with a worse outcome of EGFR-TKI treatment [4, 29, 30]. Herein, a trend towards longer OS was noted in *ROS-1* rearranged patients with strong PD-L1 expression taking crizotinib, while there was also a numerically longer OS in *KRAS*-mutant patients with strong PD-L1 expression receiving platinum double chemotherapy. By contrast, no significant impact of PD-L1 expression was observed in the outcome of *HER2*-mutant patients receiving chemotherapy. Although the case numbers were limited, we would suggest that the prognostic role of PD-L1 varies amongst different NSCLC populations.

The major limitations we encountered in this study were the retrospective nature and limited case numbers of uncommon mutations. Although data was collected retrospectively, we attempted to ensure the validity of the patients’ characteristics, the process of biomarker assessment, and the outcome measurements. Owing to the rarity of some driver mutations, we have to interpret the results with caution and it is also hard to further analyze the impact of the subtypes of each driver mutation. However, it is still worthwhile to explore more real-world data, as it may help to build up a consensus within the medical community. Therefore, further studies with larger cohorts are still required. In the present study, patients with only cytological specimens were included. All of them were prepared as cell blocks for determination of specimen adequacy and quantification of PD-L1 expression. The study by Skov BG et al. and our previous study both suggested that PD-L1 assessment is feasible on cytological material [13, 31].

## Conclusion

In conclusion, we conducted a large-scale, real-world data in order to evaluate the impact of individual driver mutations, where we suggested that different driver mutations had various influences on PD-L1 expression in NSCLC patients. Moreover, the prognostic role of PD-L1 may also be divergent amongst patients harboring different driver mutations.

## Supporting information

S1 Fig Impact of PD-L1 expression on the overall survival of advanced *KRAS*-mutant NSCLC patients receiving platinum doublet chemotherapy. (TIFF)
