# Supplementary figures and images for: Various impacts of driver mutations on the PD-L1 expression of NSCLC
Source: PLoS One. 2022 Aug 18;17(8):e0273207. doi: 10.1371/journal.pone.0273207 (PMC9387808; doi:10.1371/journal.pone.0273207)

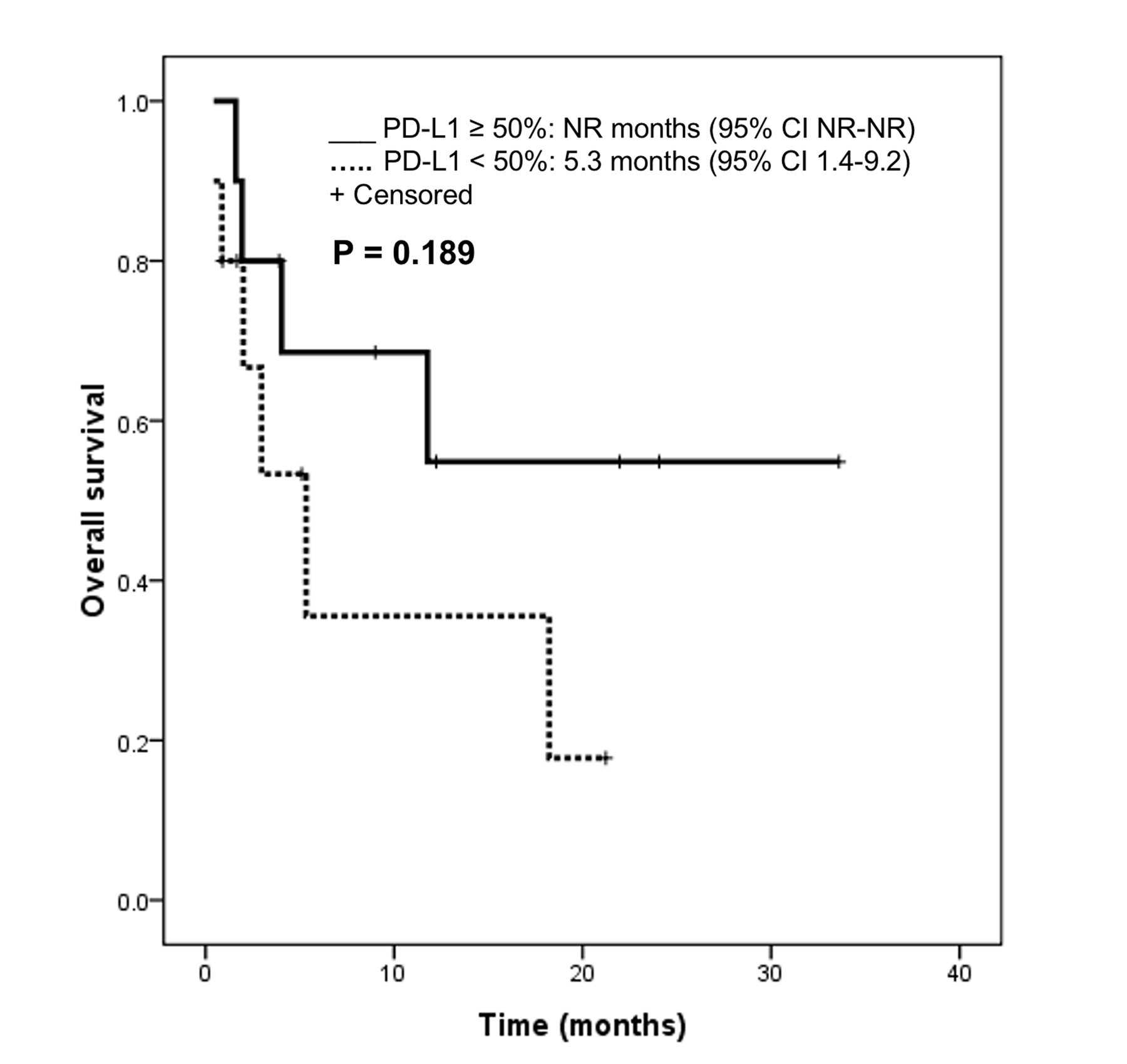

Supplement: S1 Fig — (TIFF) [file pone.0273207.s001.tiff]
